# Youth pre-pandemic executive function relates to year one COVID-19 difficulties

**DOI:** 10.3389/fpsyg.2023.1033282

**Published:** 2023-04-20

**Authors:** Alice Aizza, Blaire M. Porter, Jessica A. Church

**Affiliations:** Department of Psychology, The University of Texas at Austin, Austin, TX, United States

**Keywords:** adolescence, longitudinal, executive function, processing speed, COVID-19, mental health, socioeconomic factors, rumination

## Abstract

**Introduction:**

The first year of the COVID-19 pandemic presented a series of stressors that could relate to psychological difficulties in children and adolescents. Executive functioning (EF) supports goal achievement and is associated with life success, and better outcomes following early life adversity. EF is also strongly related to processing speed, another predictor of life outcomes.

**Methods:**

This longitudinal study examined 149 youths’ pre-pandemic EF and processing speed abilities as predictors of self-reported emotional, cognitive, and social experiences during the first year of the COVID-19 pandemic. EF and processing speed were measured with a total of 11 behavioral tasks. The COVID-era data was collected during two timepoints, during early (May-July 2020) and mid- (January-March of 2021) pandemic.

**Results:**

Better pre-pandemic EF skills and processing speed abilities predicted more mid-COVID-19 pandemic emotional and cognitive difficulties. On the other hand, better switching (a subcomponent of EF) and processing speed abilities predicted more mid-pandemic social interactions. EF and processing speed abilities did not relate to the well-being reports from the initial months of the pandemic. Our EF - but not processing speed - results were largely maintained when controlling for pre-pandemic mental health burden, socioeconomic status (SES), and gender.

**Discussion:**

Better cognitive abilities may have contributed to worse mid-pandemic functioning by supporting the meta-cognition needed for attending to the chaotic and ever-changing pandemic news and advice, leading to higher stress-induced worry and rumination. Our study highlights a potential downside of higher EF – often largely viewed as a protective factor - in youth.

## Introduction

The first year of the COVID-19 pandemic was marked by significant changes in everyone’s lives, but this was especially true for young people. The pandemic initially disrupted school routines, limited social and leisure activities, created family financial instability, and elevated global uncertainty about the virus’s spread and the future (Fegert et al., 2020). Early studies have linked lower emotional distress during the early parts of the COVID-19 pandemic with protective factors, including positive coping methods, resilience, and feelings of social connectedness (Domínguez-Álvarez et al., 2020; Magson et al., 2020; Zhang et al., 2020). Later, however, as knowledge grew with regard to COVID-19, variants came and went, and advice changed (e.g., isolation recommendations), the experienced impact of the pandemic also changed. Studies have found different patterns of change in youth internalizing problems throughout the progress of the pandemic (*cf.* Liang et al., 2021 finds a decrease in anxiety symptoms over time; Weissman et al., 2021 finds an increase in internalizing problems). The persistence of COVID-19 variants, and the pandemic’s extended duration, could contribute to change in well-being of youth that differs in patterns of impact and severity from the more universal initial stressor of the first pandemic shut-down, and thus interact differently with initially protective factors.

Several studies have identified emotional distress among children and adolescents during the COVID-19 pandemic - including symptoms of anxiety, depression, and stress - and a series of risk and protective factors associated with this distress (Duan et al., 2020; Racine et al., 2021; Tang et al., 2021). Longitudinal studies beginning before and continuing during the pandemic are important for contextualizing differences in individuals across the pandemic. For instance, Porter et al. (2021) found, through a longitudinal approach, that higher pre-pandemic ADHD symptom burden, predicted children and adolescents’ higher cognitive and emotional problems during the earliest months of the pandemic. Beyond pre-existing mental health difficulties, pre-pandemic cognitive abilities could also serve as protective or risk factors with regard to one’s functioning in the COVID-19 pandemic, and any impact could potentially vary at different points in the extended timeframe (Chahal et al., 2021).

Executive function (EF) could be an important set of cognitive abilities that support individual responses during the COVID-19 pandemic. EF refers to a collection of mental tools necessary to regulate one’s thoughts and actions, and is considered essential for the completion of goal-driven behavior (Engelhardt et al., 2019). According to the “unity and diversity” theory of EF, there is both a common EF factor across different EF skills, as well as a series of related sub-domains of EF (Friedman and Miyake, 2017). Three commonly studied sub-domains of EF are *updating*, which refers to adding new information or changing information in one’s working memory as needed; *inhibition*, referring to the suppression of a prepotent response; and *switching*, being flexible when alternating between different activities (Friedman and Miyake, 2017; Karr et al., 2018; Engelhardt et al., 2019). *Common EF* consists of the general common factor that unites these different EF abilities (Friedman and Miyake, 2017).

EF plays a role in many aspects of one’s life, including academic success and physical and mental health. Better EF abilities have been linked to higher happiness (Sung and Choi, 2021), health behaviors (Hall et al., 2008; Allan et al., 2016), emotional regulation (Groves et al., 2021), mind-wandering (Kane et al., 2007; Unsworth and Robison, 2016), resilience (Zhang et al., 2019), academic achievement (Ahmed et al., 2019), and social competence (Fong and Iarocci, 2020). Therefore, there has been evidence that higher EF abilities relate to higher well-being in youth. On the other hand, deficits in EF abilities have been associated with most psychopathologies, including major depression, conduct disorder, oppositional defiant disorder, substance use disorder, obsessive–compulsive disorder (OCD), schizophrenia, post-traumatic disorder (PTSD), and attention-deficit/hyperactivity disorder (ADHD; see Snyder et al., 2015 for review). Overall, EF consists of several mental abilities (e.g., planning, switching, inhibiting) that play a fundamental role in a person’s psychological and physical health throughout the lifespan and, consequently, could play an influential role in the well-being of young people during the COVID-19 pandemic.

Processing speed is another cognitive tool strongly related to EF (Fry and Hale, 1996). It refers to the length of time necessary to process information, formulate an appropriate reaction to it, and execute this reaction (Foong et al., 2018). Similar to EF, it is a predictor of real-world positive functioning (Puig et al., 2012), academic performance (Forchelli et al., 2021), mental health (Nigg et al., 2017; Márquez-Caraveo et al., 2021), and processing speed improves throughout the lifespan (Kail, 1991; Kail and Ferrer, 2007). Some researchers argue that it is a separate, more primitive cognitive process that aids or hinders the performance of EF, (Salthouse, 1996), while others consider it an additional EF domain (e.g., Brown et al., 2011). Thus, in addition to common EF and three EF subcomponents (inhibition, switching, and updating), we separately tested pre-pandemic measures of processing speed in relation to pandemic response.

There is a well-established link between adverse childhood experiences (ACEs) and future EF deficits (see Lund et al., 2020 for a review). ACEs refer to a wide range of negative childhood events, including household dysfunction and psychological, physical, or sexual abuse (Felitti et al., 1998). Prior research focused on EF as the mediator of later functioning among those who experienced early adversity (Kopetz et al., 2019), with higher EF abilities often serving a protective role (Tsai et al., 2020; Trossman et al., 2021). However, the direction of this relationship - whether cognitive abilities would act as protective or as risk factors to later well-being - is not clear with COVID-19 as a stressor. Early studies looking at the beginning of the COVID-19 pandemic found that higher coherence in EF-related brain networks were associated with fewer internalized symptoms (Chahal et al., 2021), and better self-reported EF abilities were associated with healthier behaviors (Appelhans et al., 2021). Subsequently, studies of mid-pandemic adults found that self-reported better attentional control abilities had a protective effect as a moderator between higher levels of COVID-19-related stress and increased anxiety (Bardeen et al., 2021), while inhibition-related prefrontal control activity predicted reduced COVID-19 distress (Monninger et al., 2022). On the other hand, better EF has also been associated with heightened worry in youth in contexts unrelated to the pandemic (Muris et al., 2002; Songco et al., 2020), so better cognitive abilities could be associated with worse pandemic functioning.

This study assessed the relationship between pre-pandemic EF and processing speed abilities, and youths’ self-reported emotional, cognitive, and social experiences during the pandemic at two different timepoints: months 3–5 and months 10–12 of year 1 of the COVID-19 pandemic (March 2020–March 2021). We hypothesized that better pre-pandemic EF and processing speed abilities (i.e., better performance in these tasks) would predict fewer emotional and cognitive difficulties, along with more social interactions during the first year of the COVID-19 pandemic (including both the early and mid-pandemic timepoints). Exploratory analyses examined change over time in those with both COVID-19 pandemic surveys, and the potential interactions of any main effects with gender, socioeconomic status, and pre-pandemic mental health.

## Methods

We analyzed data from 149 unique participants drawn from a larger sample already enrolled in an annual longitudinal study of EF abilities in youth with and without mental health difficulties (Nugiel et al., 2020). Analyzed participants had 1–4 yearly pre-pandemic data collection timepoints (in-lab) and also had provided responses to 1 or 2 online COVID-19 surveys. The pre-pandemic data points were collected between November, 2016 to March, 2020, while the first COVID-19 survey spanned May to July, 2020, and the second survey spanned January to March, 2021. The average time interval between the most recent in-lab EF visit to the first COVID-19 survey was about 13 months (*M* = 1.11 years, *range* = 0.20–3.68 years), and was about 21 months for the second COVID-19 survey time point (*M* = 1.76 years, *range* = 0.88–4.28 years).

### Participants

The 1st pandemic survey sub-sample included 135 participants (*M_age_* = 15.2 years, *SD* = 3.05, *range* = 9.45–22.1), with 61 identifying as female, 70 as male, 3 as non-binary, and 1 ‘preferred not to say’. The sub-sample for the second COVID-19 timepoint included 107 participants (*M_age_* = 15.7 years, *SD* = 3.02, *range* = 10.1–21.7), of which 52 identified as females, 51 as males, 2 as non-binary, and 2 ‘preferred not to say’. There were 93 overlapping participants between the two COVID-19 sub-samples. Participants who identified as non-binary or preferred not to report gender were removed from the gender-related subanalysis for early- (yielding *n* = 131) and mid-pandemic (yielding *n* = 103) timepoints.

Across all samples, the majority identified as white, and a substantial number of participants had a mental health diagnosis, with ADHD (with and without comorbidity) being the most common. See Supplementary Table S1 for the complete demographic information for each subsample, including race/ethnicity, income, diagnosis, and medication use.

All data collection was reviewed and approved by the University of Texas at Austin Institutional Review Board. Parents of children younger than 18 years of age provided informed consent while children provided assent before each data collection time point. Adult participants provided informed consent before each data collection time point. After the completion of each session, both the parent (if present due to the child being <18 years) and youth participant received financial compensation.

### Measures and procedures

#### Pre-pandemic data collection

The annual pre-pandemic EF study data was collected through a 3-hour in-person lab session that included the EF and processing speed tasks. Participants and their parent/legal guardian completed a series of questionnaires, in addition to the youth participant completing the behavioral data collection.

We administered 8 EF and 3 processing speed tasks, adapted from (Engelhardt et al., 2015). See Supplementary Table S2 for task description and scoring, and Engelhardt et al. (2015) for reliability and factor analysis of the tasks. All task scores were z-scored among the whole (*n* = 149) sample’s most-recent pre-pandemic data. Task data were excluded if the participant was greater than 3.5 standard deviations from the average, which excluded 1 Cognitive Flexibility score and 2 N-Back scores. All eligible EF task scores were averaged within each putative EF domain to compose their respective scores (see Supplementary Table S2). Common EF was calculated as a composite score of the mean of the inhibition, updating, and switching variables. Processing speed was calculated as the mean of the three processing speed tasks, similarly z-scored. One participant’s score was greater than 3.5 standard deviations and removed. No participant was removed from the sample.

Pre-pandemic mental health burden was measured with the parent-reported Child Behavior Checklist (CBCL; Achenbach and Rescorla, 2001) among those under 18 years old, and self-reported ASR (Achenbach and Rescorla, 2003) for those older than 18 (*n* = 16). The two measures were converted to percent-of-max score (POMS) - the participant score minus the measure’s minimum possible score, all divided by the measure’s maximum score minus the minimum score in order to combine across the two measures.

#### COVID-19 mental wellbeing assessment

The data collected during the COVID-19 pandemic occurred via online survey. The participants and their parents (if younger than 18) were contacted through email, phone call, and/or text message. If interested, participants were compensated with a $5 Amazon card for each survey completed. Youth experiences during the pandemic were measured with the COVID-19 Adolescent Symptom and Psychological Experience Questionnaire (CASPE); this analysis just included questions about emotional, cognitive, and social experiences during the pandemic (Ladouceur, 2020). For instance, participants were asked to rate how ‘stressful’ they perceived the pandemic’s uncertainty through a five-point Likert scale ranging from “very slightly or not at all” to “extremely.” This data set was coded as in Porter et al. (2021), so that a higher score on the emotional and cognitive variables represented more difficulties in these dimensions, while a higher score on the social variable represented more frequent online social interactions. The points from each question were summed to compose the emotional, social, and cognitive variables scores. Subsequently, the three variable scores were converted to POMS and z-scored. Porter et al. (2021) calculated the early-pandemic Cronbach’s alpha for the CASPE, while the mid-pandemic Chronbach’s alpha can be found at Supplementary Table S3.

#### Demographic characteristics

Participant date of birth was used to calculate age during the EF in-person sessions and COVID-19 survey completions. Gender was collected in the first COVID-19 survey but not in the second one. Therefore, this study included the most recent reported gender from the first COVID-19 timepoint and, if not available, the first EF data timepoint. Data collection from the first EF data timepoint included race and ethnicity, while each annual visit collected diagnoses, medication status, income, and parental education information. The socioeconomic status measure used in this analysis was a composite measure of z-scored income and parental (including both parental figure’s) education.

### Statistical analysis

Data were analyzed with R-4.1.1 (R Core Team, 2021). Unless noted otherwise, age at the respective COVID-19 time point was added as a covariate, and all value of *p*s were adjusted using the False Discovery Rate (FDR) correction (Benjamini and Hochberg, 1995).

#### Pre-pandemic cognitive abilities as COVID-19-era predictors

Multiple linear regressions were conducted using common EF, a single EF subcomponent (inhibition, updating, and switching were separately tested), or processing speed scores as a predictor variable and one of the COVID-19 social, cognitive, and emotional experience scores as the outcome variable (3 tests per predictor variable). All analyses were separately performed for each COVID-19 timepoint, first (May–July 2020) and second (January–March 2021). We subsequently controlled for family ties within our sample by bootstrapping a random individual per family (*n* = 68 at COVID timepoint 2) one-thousand times and obtained the subgroup mean *p*-value of the primary significant whole group models.

#### Additional exploratory analyses

Pre-pandemic mental health symptom burden, gender, socioeconomic composite measures, and the time interval between the respective pre-pandemic EF collection and COVID survey data were added to the regression models to see whether they affected the significant EF and processing speed results. These covariates were examined separately as predictors of COVID-era functioning, along with age, if not previously examined by Porter et al. (2021). Data from participants with both COVID-19 timepoints (*n* = 93) were tested to explore whether the two timepoints’ emotional, cognitive, and social variables differed within-person across the pandemic via within-subject *t*-tests.

## Results

### Pre-pandemic EF and processing speed abilities as a COVID-19-era predictor

We hypothesized that better EF and processing speed abilities would predict better functioning in both timepoints. We first tested whether common EF, and its subdomains of inhibition, updating, and switching, were predictors of early pandemic (May–July 2020) emotional, cognitive, and social functioning in youths. Counter to the hypothesis, there was no relationship between any EF or processing speed variables and any aspect of early pandemic functioning (Supplementary Table S4).

**Figure 1:**
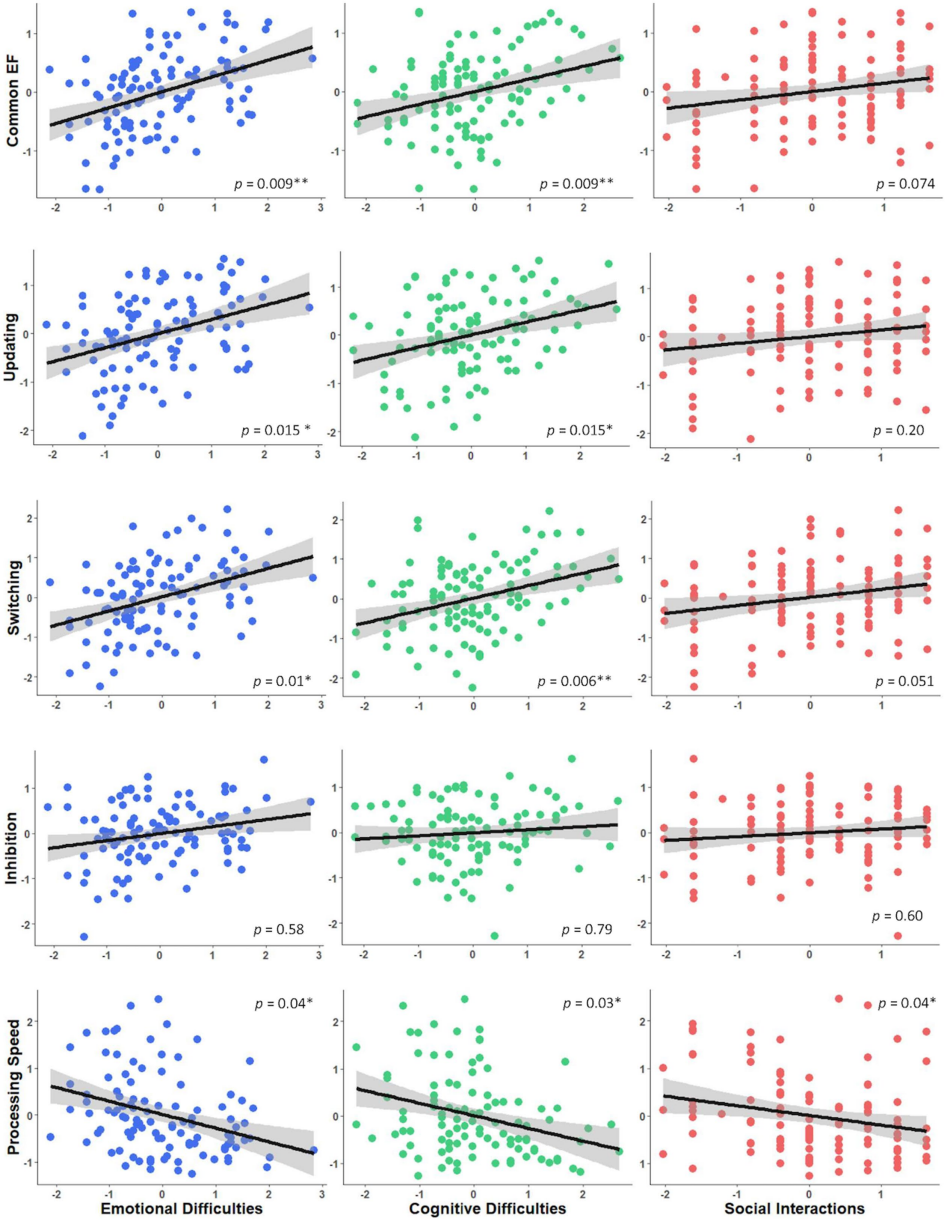
EF and processing speed as predictors of mid-pandemic functioning. Common EF, and subdomains of inhibition, updating, and switching as predictors of higher social, and worse cognitive and emotional experiences at the COVID-19 pandemic second time point survey (January–March 2021). The relationships are displayed without age regression, while all reported statistics had age as a covariate. Higher processing speed scores reflect slower speeds. The y-axis labels (e.g., updating) refer to the predictor measures across the entire respective row, while the x-axis labels (e.g., Emotional Difficulties) apply the outcomes for all graphs in that respective column. **p < 0.01, *p < 0.05, all FDR corrected.

We next assessed whether common EF or any of its three subdomains predicted social, cognitive, and emotional experiences in youths mid-pandemic (January–March 2021). We found significant relations with EF and processing speed and pandemic self-reports at this time point, but they were not in the predicted direction (Figure 1). Pre-pandemic common EF (*β* = 0.53, *p* = 0.009, *R*^2^ = 0.17, *Adjusted R*^2^ = 0.15), updating (*β* = 0.34, *p* = 0.015, *R*^2^ = 0.16, *Adjusted R*^2^ = 0.14), switching (*β* = 0.37, *p* = 0.01, *R*^2^ = 0.16, *Adjusted R*^2^ = 0.15), and processing speed (*β* = −0.28, *p* = 0.04, *R*^2^ = 0.13, *Adjusted R*^2^ = 0.11) abilities were moderate-to-strongly related to mid-pandemic emotional difficulties, such that better cognitive abilities predicted more emotional difficulties. Better pre-pandemic common EF (*β* = 0.52, p = 0.009, *R*^2^ = 0.11, *Adjusted R*^2^ = 0.089), updating (*β* = 0.38, *p* = 0.015, *R*^2^ = 0.11, *Adjusted R*^2^ = 0.092), switching (*β* = 0.44, *p* = 0.006, *R*^2^ = 0.12, *Adjusted R*^2^ = 0.11), and processing speed (*β* = −0.36, *p* = 0.03, *R*^2^ = 0.098, *Adjusted R*^2^ *=* 0.81) abilities were also moderate-to-strongly significant predictors of more mid-pandemic cognitive difficulties. Processing speed (*β* = −0.32, *p* = 0.04, *R*^2^ = 0.058, *Adjusted R*^2^ = 0.039) and switching (*β* = 0.28, *p* = 0.051, *R*^2^ = 0.052, *Adjusted R*^2^ = 0.034, marginal), were the only weak significant predictors of social interaction, such that better cognitive abilities predicted more frequent online interactions. Common EF and updating were not predictors of social interactions. Inhibition was not related to any CASPE emotional, cognitive, and social functioning (Figure 1). See Supplementary Table S4 for non-significant results.

As several families had multiple children included in the full mid-pandemic sample (*n* = 68 unique families, with *n* = 107 data points), we explored whether using single data points from families altered the relationship between EF and processing speed with mid-pandemic functioning. When generating a mean value of p from bootstrapping a random individual per family (*n* = 68) one-thousand times, within the mid-pandemic subsample, common EF still marginally predicted emotional functioning, but not cognitive and social functioning. Updating remained a significant predictor of mid-pandemic emotional and cognitive difficulties. However, switching and processing speed became non-significant predictors of mid-pandemic cognitive and social functioning. See Supplementary Table S5 for these results.

### Additional exploratory analyses

#### COVID-19 timepoint-to-timepoint change

**Figure 2:**
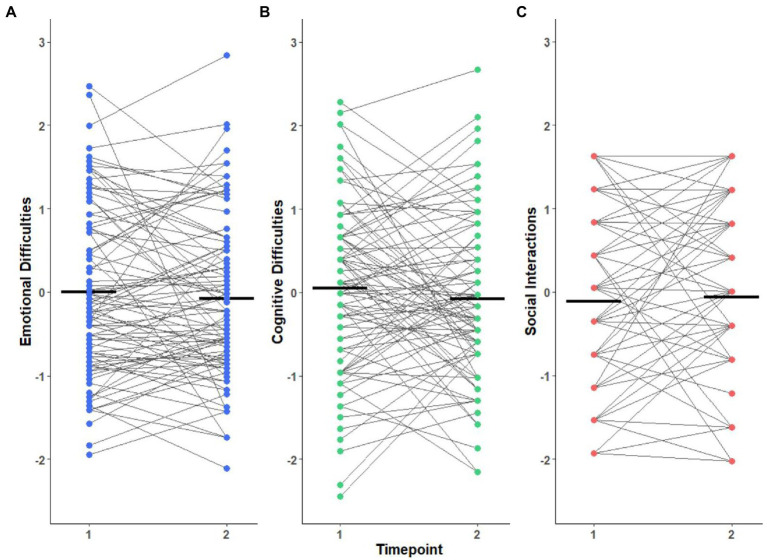
Early and Mid-COVID-19-Era experience change. Individual change in CASPE scores between early and mid-pandemic collection timepoints (*n* = 93). **(A)** Emotional difficulties, **(B)** Cognitive difficulties, and **(C)** Social interactions. Points represent individual participants, with thin lines connecting each participant’s early and mid-pandemic experiences. Bolded horizontal lines indicate group mean within each timepoint. Means were not significantly different, but this masked considerable individual variability. The x-axis (timepoint) is the same across all three graphs.

For participants who completed both COVID-19 timepoint surveys, a within-subjects t-test was used to evaluate whether early- and mid-pandemic social, cognitive, and emotional experience differed (*n* = 93). As shown in Figure 2, there were no significant mean differences between early and mid-pandemic emotional (*t*(92) = 0.88, *p* = 0.57), cognitive (*t*(92) = 1.22, *p* = 0.57), and social (*t*(92) = −0.52, *p* = 0.60) functioning, although there was great variability across individuals.

#### Gender

We first tested whether there were gender differences in the pandemic survey responses about early- and mid-pandemic social, cognitive, and emotional functioning. There was a significant gender difference in emotional experience at both the first (*t*(129) = 4.07, *p* = 0.00025) and second (*t*(101) = 2.60, *p* = 0.032) survey time points after FDR-correction, such that males reported better emotional functioning than females during both time points. There were no significant gender differences in cognitive and social functioning within either time point (Supplementary Table S6).

Next, we controlled for gender in our main results. In general, controlling for gender did not alter relations between pre-pandemic EF abilities and mid-pandemic emotional and cognitive functioning (Supplementary Table S6). However, after adding the control for gender, switching (*β* = 0.21, *p* = 0.15, *R*^2^ = 0.058, *Adjusted R*^2^ = 0.029) became a non-significant predictor of mid-pandemic social interactions. Similarly, once gender was added, processing speed became a non-significant predictor of emotional difficulties (*β* = −0.22, *p* = 0.11, *R*^2^ = 0.19, *Adjusted R*^2^ = 0.16) and social interactions (*β* = −0.26, *p* = 0.10, *R*^2^ = 0.061, *Adjusted R*^2^ = 0.032), while marginally predicting cognitive functioning, *β* = −0.33, *p* = 0.053, *R*^2^ = 0.095, *Adjusted R*^2^ = 0.067.

#### Socioeconomic status

As for gender, we first tested whether the most recent pre-pandemic socioeconomic composite measure was a predictor of youth experiences during the two COVID-19 surveys. Pre-pandemic socioeconomic status did not predict youth early- or mid-pandemic emotional, cognitive, and social functioning (see Supplementary Table S6). Next, controlling for age and socioeconomic status did not alter the original results between common EF, updating, switching, or processing speed, and mid-COVID-19 pandemic functioning (see Supplementary Table S7 for complete results).

#### Mental health burden

Pre-pandemic mental health burden of youth, as rated by parents, did not predict emotional, cognitive, and social functioning in early pandemic (as found in Porter et al., 2021), nor mid-pandemic (Supplementary Table S6). Controlling for age and mental health burden did not alter in direction or magnitude the common EF, updating, switching, and processing speed main results (Supplementary Table S7).

#### Time interval

The time interval between youth pre-pandemic and COVID-era assessments did not predict emotional, cognitive, and social functioning in either early pandemic or mid-pandemic (Supplementary Table S6). Controlling for age and time period did not alter in direction or magnitude the common EF, updating, switching, and processing speed main results (Supplementary Table S7).

## Discussion

### Mid-pandemic outcomes worsened with higher cognitive abilities

It was hypothesized that better pre-pandemic EF and processing speed abilities would have a protective effect against emotional and cognitive difficulties during the two COVID-19 timepoints. However, instead we found no effect on early COVID-19 experiences, and we found that better cognitive abilities actually predicted worse mid-pandemic functioning. More specifically, stronger common EF, updating, switching, and processing speed abilities significantly predicted worse youth emotional and cognitive functioning in early 2021. We consider these findings in the context of existing literature on rumination, worry, and pre-pandemic quality of life.

Our findings suggest a potential relationship between more advanced cognitive development and more severe pandemic worry in youth. Perica and colleagues found that higher pre-pandemic hippocampal-prefrontal connectivity predicted higher mid-pandemic anxiety among 10-19-year-old individuals (Perica et al., 2021). These brain networks have been associated with memory formation, emotional processing, and high-order cognitive processing, including decision-making and planning abilities (Calabro et al., 2019). Based on these results, the authors hypothesized that adult-like cognitive abilities support future thinking that promotes stress-induced worry (Perica et al., 2021). EFs are higher-order mental processes that support this type of cognitive activity (Kopetz et al., 2019). Additionally, Muris et al. (2002) found a positive association between cognitive maturity (as assessed by Piaget conservation tasks) and worry among youth. These cognitive abilities are hypothesized to promote and enable thinking about future events and anticipating adverse outcomes, especially among older children and adolescents (Muris et al., 2002; Songco et al., 2020). Therefore, more mature cognitive abilities could have worsened mid-pandemic cognitive and emotional functioning through heightened worry by supporting planning, meta-cognition, and future-focused thinking.

The relationship between better cognitive abilities and worse emotional and cognitive functioning are also consistent with the biocognitive vulnerability-stress model. This model describes how normative adolescent brain and EF development are necessary to have the negative cognitive style (i.e., negatively interpreting life events) often seen in those with depression (Alloy and Abramson, 2007). Two processes could mediate the association between cognitive development and negative affect: First, cognitive resources would be required to evaluate hypothetical undesirable consequences of current stressful events, leading to the feeling of hopelessness; Second, updating is hypothesized to be especially fundamental for the development of rumination and self-regulation due to its role in keeping information in one’s mind (Alloy and Abramson, 2007; Altamirano et al., 2010; Zetsche and Joormann, 2011; Wagner et al., 2014). Our study’s findings are remarkably consistent with this model: the subdomain of updating played a major role in the relationship between EF and mid-COVID-19 functioning. Thus, we propose that better cognitive abilities could have acted as prerequisites for rumination and negative interpretations of the stress-inducing pandemic, and, consequently, related to worse mid-COVID-19 pandemic emotional and cognitive experiences.

Alternatively, these findings could be reflecting better pre-pandemic overall quality of life in those with better cognitive skills. Better EF and processing speed has been associated with higher extraversion (Campbell et al., 2011), optimal school performance (Duncan et al., 2007; Mulder et al., 2010) and quality of life (Brown and Landgraf, 2010; Ojeda et al., 2012; Zhang et al., 2021). Therefore, individuals with better pre-pandemic EF could have experienced more enjoyment out of social and academic experiences that were largely still unavailable mid-pandemic in our sample, including in-person peer interaction, school engagement, and quality time at school. The loss of these experiences could have contributed to worse mid-pandemic emotional and cognitive functioning, as that survey was collected halfway through the unusual school year of 2020–2021.

There was strong agreement between EF and processing speed results. Processing speed is highly correlated with EF abilities, and, consequently, better processing speed could relate to worse pandemic emotional and cognitive functioning through similar mechanisms as discussed above. These findings are consistent with previous literature describing the strong link between EF and processing speed (Fry and Hale, 1996). Processing speed and EF are not always collected within the same datasets, and thus our study presented an important opportunity to examine them both within the same individuals. The similar, if weaker, impact of processing speed on mid-pandemic results indicate that overall better cognitive functioning may relate to worse mid-pandemic cognitive and emotional outcomes.

#### Higher cognitive abilities increased online social interactions

Processing speed and switching (marginally) showed a protective role on mid-pandemic social functioning, as better cognitive abilities predicted more frequent online social interactions. Previous studies have shown a similar protective effect of processing speed (Bachman et al., 2011) and switching (Bock et al., 2014) within social interactions among adolescents. The first year of the COVID-19 pandemic was marked by social distancing guidelines, which may have limited children and adolescents’ in-person opportunities for social interaction. Within this context, our results suggest that better switching and processing speed could facilitate adaptation from this shift from in-person to online means of interacting with others. Both switching and processing speed have been previously associated with adaptability (Bertollo et al., 2019; Thornton et al., 2021). Therefore, better processing speed - and switching, although not as strongly - could relate to better mid-pandemic social functioning through more optimal adaptation.

#### We found no early pandemic effects

It is noteworthy that we found no relationship between pre-pandemic EF, EF subdomains (updating, switching, and inhibition), or processing speed with early COVID-19 functioning. Our results underscore the importance of timing in pandemic surveys, suggesting distinctions between the early and mid-pandemic periods. The initial months of the pandemic – including May to July of 2020 – were marked by global uncertainty and fear about COVID-19. On the other hand, the pandemic’s health, safety, and social distancing guidelines had changed up and down for several months by the second COVID-19 survey timepoint (January–March, 2021). It is possible that the early months of the pandemic - times of more universal uncertainty and consistent shutdown - provided a context where differences related to cognitive abilities were minimized, because impact was more similar. Subsequently, however, cognitive abilities could have been more critical when there was more information available about the COVID-19 as a virus, more discussion of mixed or negative future projections, and there was more variation in pandemic response at different levels (e.g., city, school, family). The information needed for future-oriented thinking could have contributed to more worry and rumination among those with better cognitive abilities at this later timepoint, consistent with the large variability seen across individuals between survey timepoints.

Metacognition (i.e., awareness of self-cognitive processes) could also explain timepoint differences in self-reported emotional and cognitive experience. A portion of the CASPE emotional and cognitive difficulties questions inquired about individuals’ mental states in comparison to before the COVID-19 pandemic (e.g., “Compared to before the COVID-19 outbreak”) instead of at the current week (e.g., “In the past 7 days”; Ladouceur, 2020). Better long-term recollection of cognitive state has been associated with better metacognition, and also with stronger cognitive abilities (see Roebers, 2017, for a review). Therefore, the association between better cognitive abilities and self-reported worse emotional and cognitive experience could be mediated by metacognition abilities. These metacognition abilities would be more strongly needed for mid-pandemic retrieval compared to early-pandemic, due to the longer time interval between the pre-COVID-19 period and survey completion. This additional cognitive demand could also have mediated EF and processing speed as better predictors of mid-pandemic functioning instead of early pandemic functioning.

#### Gender related to pandemic emotional response and social interactions

Girls were found to experience worse emotional functioning than boys early- and mid-pandemic. These findings are consistent with other studies that found higher rates of internalized symptoms among female youth than males during early (Duan et al., 2020; Zhou et al., 2020) and mid-pandemic periods (Perica et al., 2021). This pattern could reflect increased stress vulnerability among females (Natsuaki et al., 2009) or normative higher depression and anxiety rates among girls outside of the context of the pandemic (e.g., Alloy and Abramson, 2007). However, these results were independent of our EF results, suggesting a somewhat separate phenomenon impacting reports of pandemic well-being, and an effect limited to emotional responses, rather than cognitive responses.

When controlling for gender, processing speed’s relationship with emotional and social functioning became non-significant. Similarly, the relationship between switching and social interactions weakened when controlling for gender. These results indicate a potential gender difference within EF subdomains and processing speed as predictors of mid-pandemic COVID-era functioning, particularly in the social aspect, despite no overall gender difference in processing speed or switching.

#### Pre-pandemic mental health, socioeconomic status, and assessment time interval did not impact results

Common EF, updating, switching, and processing speed results remained largely unaltered when separately controlling for pre-pandemic mental health burden, socioeconomic status, and the time interval between assessments. These results indicate that cognitive ability predicted pandemic emotional and cognitive functioning across important differences in individual experience. Controlling for these variables was particularly important considering previous evidence of the relationship between EF and processing speed with socioeconomic status (Hackman et al., 2015; Madhushanthi et al., 2018), mental health burden (Robson et al., 2020), and most psychopathologies (Snyder et al., 2015; Gur et al., 2019). Also, these pre-pandemic covariates were particularly important to examine in this sample, as 54% of participants had at least one mental health diagnosis, with ADHD being the most common diagnosis (80% of diagnosed). Despite considerable variability in the timing between our pre-pandemic assessment and the COVID surveys, our results suggest that the results were robust to this variability.

### Strengths and limitations

The collection of multiple EF and processing speed measures within a relatively large sample of adolescents is a strength of the current study, compensating for possible noise within each task. In addition, the two COVID-19 timepoints allowed for a more complete picture of the first year of the COVID-19 pandemic, which is especially important as COVID-19 has been a fast-changing global event. Also, the richness of the pre-pandemic data collection, with the inclusion of several pre-pandemic measures, allowed exploration of important covariates such as socioeconomic status and mental health, and highlighted the consistency of the present results. Further, including both diagnosed and undiagnosed youth longitudinally broadens the impact of our results across more children’s experiences.

The present study also has several limitations. The cognitive ability measures (EF and processing speed) were only administered before the COVID-19 pandemic, not during. Administering a self-reported EF measure such as the questionnaire Behavior Rating Inventory of Executive Function (BRIEF; Gioia et al., 2000) during the pandemic could have been one way to test the association between simultaneous EF and COVID-19 well-being, although previous studies have shown limited agreement between questionnaire-based and performance-based EF (Toplak et al., 2012). Although we controlled for age within all models, our findings should be interpreted with caution as our sample had a large age range (from 9.45–22.1 years old), and thus there may be important variations in pandemic response by age that are important to consider in a larger sample. Similarly, direct comparisons of social, cognitive, and emotional variables before and during the COVID-19 pandemic were not possible as in-person collection was not possible during these periods of the pandemic, and the CASPE was created because of the COVID-19 pandemic (Ladouceur, 2020). However, a different pre-pandemic mental health burden measure was included and explored as a covariate (i.e., the CBCL; Achenbach and Rescorla, 2001), and did not impact our results.

Further, a substantial portion of the youth in our study sample had at least one mental health diagnosis, and this is an important point to consider regarding the generalizability of our findings. It is also important to note that up to 1 in 5 children experience a mental health diagnosis and mental health difficulties (Child Mental Health | CDC, 2020). Therefore, by including children with and without mental health difficulties, we may be increasing the applicability or utility of our results, at least in the context of mental health.

Unfortunately, our EF and processing speed results were not fully robust to the power hit of reducing data points to one child per family (from *n* = 107 to *n* = 68). The shared variance by family members thus may have contributed to our effects, and family-level dynamics are worthy of future exploration. Lastly, the social functioning measure evaluated only the frequency of online social interactions, which might not directly reflect other important qualitative and quantitative aspects of these social interactions.

## Conclusion

These findings indicate that stronger pre-pandemic cognitive abilities – assessed via aspects of EF and processing speed - predicted more emotional and cognitive difficulties mid-pandemic, but not during the first few months of the COVID-19 pandemic. These findings suggest that stronger pre-pandemic cognitive abilities could have promoted greater stress-induced rumination and worry among youth during lingering aspects of this global, constantly shifting, pandemic experience. Processing speed and switching abilities were also shown to have a beneficial role in more frequent mid-pandemic social interaction. Our findings could help to identify and aid youth at risk for negative outcomes associated with the long-lasting and unpredictable COVID-19 pandemic.

Future research could benefit from exploring elements that mediate the association between EF and mental health. For instance, it could be the case that higher exposure to negative information results in higher anxiety among youth with higher EF and processing speed abilities, or it could be the uncertainty from mixed messaging by societal leaders, or the sensitivity to stress of adults in the child’s environment. Similarly, researchers could explore further coping strategies to attenuate negative affective and cognitive difficulties among youth with higher cognitive abilities.

Our findings bring attention to the potential downside of higher EF and cognitive skills in youth - greater awareness of the world can have its negatives during times of prolonged upheaval and mixed messaging. Youth with higher cognitive abilities, often viewed as at lower risk for emotional and cognitive distress, are possibly in need of specific treatment and support when in a prolonged and unpredictable pandemic context. Support could include helping youth interpret media messaging, helping them understand how to evaluate and contextualize their own circumstances relative to bigger national or international issues, and helping them to gather information or avoid disinformation as events unfold and change over time.

## Data availability statement

The raw data supporting the conclusions of this article will be made available by the authors, without undue reservation.

## Ethics statement

The studies involving human participants were reviewed and approved by University of Texas at Austin IRB. Written informed consent to participate in this study was provided by the participants’ legal guardian.

## Author contributions

AA and JC conceived the research question. AA ran the analyses and wrote the first draft. BP and JC assisted with analysis and edited the manuscript. All authors contributed to the article and approved the submitted version.

## Funding

This study was funded by the Brain and Behavior Research Foundation NARSAD Young Investigator Award (JC), and University of Texas funds (JC).

## Conflict of interest

The authors declare that the research was conducted in the absence of any commercial or financial relationships that could be construed as a potential conflict of interest.

## Publisher’s note

All claims expressed in this article are solely those of the authors and do not necessarily represent those of their affiliated organizations, or those of the publisher, the editors and the reviewers. Any product that may be evaluated in this article, or claim that may be made by its manufacturer, is not guaranteed or endorsed by the publisher.

## References

[ref1] Achenbach T.M. Rescorla L.A. (2001). Manual for the ASEBA School-Age Forms & Profiles. Burlington, VT: University of Vermont, Research Center for Children, Youth, and Families.

[ref2] Achenbach T.M. Rescorla L.A. (2003). Manual for the ASEBA Adult Forms & Profiles. Burlington, VT: University of Vermont, Research Center for Children, Youth, and Families.

[ref3] Ahmed S. F. Tang S. Waters N. E. Davis-Kean P. (2019). Executive function and academic achievement: longitudinal relations from early childhood to adolescence. J. Educ. Psychol. 111, 446–458. doi: 10.1037/edu0000296

[ref4] Allan J. L. McMinn D. Daly M. (2016). A bidirectional relationship between executive function and health behavior: evidence, implications, and future directions. Front. Neurosci. 10:386. doi: 10.3389/fnins.2016.00386, PMID: 27601977 PMC4993812

[ref5] Alloy L. B. Abramson L. Y. (2007). “The adolescent surge in depression and emergence of gender differences: a biocognitive vulnerability-stress model in developmental context,” in Adolescent Psychopathology and the Developing Brain: Integrating Brain and Prevention Science. eds. Romer D. Walker E. F. (New York, NY: Oxford University Press), 284–312.

[ref6] Altamirano L. J. Miyake A. Whitmer A. J. (2010). When mental inflexibility facilitates executive control. Psychol. Sci. 21, 1377–1382. doi: 10.1177/0956797610381505, PMID: 20798398 PMC4323352

[ref7] Appelhans B. M. Thomas A. S. Roisman G. I. Booth-LaForce C. Bleil M. E. (2021). Preexisting executive function deficits and change in health behaviors during the COVID-19 pandemic. Int. J. Behav. Med. 28, 813–819. doi: 10.1007/s12529-021-09974-0, PMID: 33649889 PMC7920747

[ref8] Bachman P. Niendam T. A. Jalbrzikowkski M. Park C. Y. Daley M. Cannon T. D.. (2011). Processing speed and neurodevelopment in adolescent-onset psychosis: cognitive slowing predicts social function. J. Abnorm. Child Psychol. 40, 645–654. doi: 10.1007/s10802-011-9592-5, PMID: 22134489 PMC4119229

[ref9] Bardeen J. R. Gorday J. Y. Clauss K. (2021). The moderating effect of attentional control on the relationship between COVID stress and generalized anxiety symptoms. Psychol. Rep. 125, 2517–2530. doi: 10.1177/00332941211025260, PMID: 34120535

[ref10] Benjamini Y. Hochberg Y. (1995). Controlling the false discovery rate: a practical and powerful approach to multiple testing. J R Stat Soc Ser B 57, 289–300. doi: 10.1111/j.2517-6161.1995.tb02031.x

[ref11] Bertollo J. R. Strang J. F. Anthony L. G. Kenworthy L. Wallace G. L. Yerys B. E. (2019). Adaptive behavior in youth with autism Spectrum disorder: the role of flexibility. J. Autism Dev. Disord. 50, 42–50. doi: 10.1007/s10803-019-04220-9, PMID: 31520244

[ref12] Bock A. M. Gallaway K. C. Hund A. M. (2014). Specifying links between executive functioning and theory of mind during middle childhood: cognitive flexibility predicts social understanding. J. Cogn. Dev. 16, 509–521. doi: 10.1080/15248372.2014.888350

[ref14] Brown T. E. Landgraf J. M. (2010). Improvements in executive function correlate with enhanced performance and functioning and health-related quality of life: evidence from 2 large, double-blind, randomized, placebo-controlled trials in ADHD. Postgrad Med 122, 42–51. doi: 10.3810/pgm.2010.09.2200, PMID: 20861587

[ref15] Brown T. E. Reichel P. C. Quinlan D. M. (2011). Executive function impairments in high IQ children and adolescents with ADHD. Open J Psychiatry 1, 56–65. doi: 10.4236/ojpsych.2011.12009

[ref16] Calabro F. J. Murty V. P. Jalbrzikowski M. Tervo-Clemmens B. Luna B. (2019). Development of hippocampal–prefrontal cortex interactions through adolescence. Cereb. Cortex 30, 1548–1558. doi: 10.1093/cercor/bhz186, PMID: 31670797 PMC7132933

[ref001] Campbell A. M. Davalos D. B. McCabe D. P. Troup L. J. (2011). Executive functions and extraversion. Pers. Individ. Differ. 51, 720–725. doi: 10.1016/j.paid.2011.06.018, PMID: 29372603

[ref17] Chahal R. Kirshenbaum J. S. Miller J. G. Ho T. C. Gotlib I. H. (2021). Higher executive control network coherence buffers against puberty-related increases in internalizing symptoms during the COVID-19 pandemic. Biol Psychiatry Cogn Neurosci Neuroimaging 6, 79–88. doi: 10.1016/j.bpsc.2020.08.010, PMID: 33097469 PMC7455201

[ref18] Child Mental Health | CDC. (2020). Centers for Disease Control and Prevention. Available at: https://www.cdc.gov/childrensmentalhealth/features/child-mental-health.html (Accessed October 13, 2022).

[ref19] Domínguez-Álvarez B. López-Romero L. Isdahl-Troye A. Gómez-Fraguela J. A. Romero E. (2020). Children coping, contextual risk and their interplay during the COVID-19 pandemic: a Spanish case. Front. Psychol. 11:3427. doi: 10.3389/fpsyg.2020.577763, PMID: 33391095 PMC7772313

[ref20] Duan L. Shao X. Wang Y. Huang Y. Miao J. Yang X.. (2020). An investigation of mental health status of children and adolescents in China during the outbreak of COVID-19. J. Affect. Disord. 275, 112–118. doi: 10.1016/j.jad.2020.06.029, PMID: 32658812 PMC7329661

[ref002] Duncan G. J. Dowsett C. J. Claessens A. Magnuson K. Huston A. C. Klebanov P.. (2007). School readiness and later achievement. Dev. Psychol. 43, 1428–1446. doi: 10.1037/0012-1649.43.6.1428, PMID: 18020822

[ref21] Engelhardt L. E. Briley D. A. Mann F. D. Harden K. P. Tucker-Drob E. M. (2015). Genes unite executive functions in childhood. Psychol. Sci. 26, 1151–1163. doi: 10.1177/0956797615577209, PMID: 26246520 PMC4530525

[ref22] Engelhardt L. E. Harden K. P. Tucker-Drob E. M. Church J. A. (2019). The neura architecture of executive functions is established by middle childhood. NeuroImage 185, 479–489. doi: 10.1016/j.neuroimage.2018.10.024, PMID: 30312810 PMC6366457

[ref23] Fegert J. M. Vitiello B. Plener P. L. Clemens V. (2020). Challenges and burden of the coronavirus 2019 (COVID-19) pandemic for child and adolescent mental health: a narrative review to highlight clinical and research needs in the acute phase and the long return to normality. Child Adolesc Psychiatry Ment Health 14:20. doi: 10.1186/s13034-020-00329-3, PMID: 32419840 PMC7216870

[ref24] Felitti V. J. Anda R. F. Nordenberg D. Williamson D. F. Spitz A. M. Edwards V.. (1998). Relationship of childhood abuse and household dysfunction to many of the leading causes of death in adults. Am. J. Prev. Med. 14, 245–258. doi: 10.1016/s0749-3797(98)00017-8, PMID: 9635069

[ref25] Fong V. C. Iarocci G. (2020). The role of executive functioning in predicting social competence in children with and without autism Spectrum disorder. Autism Res. 13, 1856–1866. doi: 10.1002/aur.2350, PMID: 33460309

[ref26] Foong H. F. Hamid T. A. Ibrahim R. Haron S. A. (2018). Information processing speed as a mediator between psychosocial stress and global cognition in older adults. Psychogeriatrics 18, 21–29. doi: 10.1111/psyg.12279, PMID: 29372603

[ref27] Forchelli G. A. Vuijk P. J. Colvin M. K. Ward A. K. Koven M. R. Dews A.. (2021). What is a processing speed weakness? Importance of cognitive ability when defining processing speed in a child psychiatric population. Child Neuropsychol. 28, 266–286. doi: 10.1080/09297049.2021.1972957, PMID: 34544318 PMC9284538

[ref28] Friedman N. P. Miyake A. (2017). Unity and diversity of executive functions: individual differences as a window on cognitive structure. Cortex 86, 186–204. doi: 10.1016/j.cortex.2016.04.023, PMID: 27251123 PMC5104682

[ref29] Fry A. F. Hale S. (1996). Processing speed, working memory, and fluid intelligence: evidence for a developmental Cascade. Psychol. Sci. 7, 237–241. doi: 10.1111/j.1467-9280.1996.tb00366.x

[ref30] Gioia G. A. Isquith P. K. Guy S. C. Kenworthy L. (2000). Behavior Rating Inventory of Executive Function (BRIEF). Odessa, FL: PAR Psychological Assessment Resources, Inc.

[ref31] Groves N. B. Wells E. L. Soto E. F. Marsh C. L. Jaisle E. M. Harvey T. K.. (2021). Executive functioning and emotion regulation in children with and without ADHD. Res Child Adolesc Psychopathol 50, 721–735. doi: 10.1007/s10802-021-00883-0, PMID: 34762251 PMC9091051

[ref005] Gur R. E. Moore T. M. Rosen A. F. Barzilay R. Roalf D. R. Calkins M. E.. (2019). Burden of environmental adversity associated with psychopathology, maturation, and brain behavior parameters in youths. JAMA Psychiatry 76, 966. doi: 10.1001/jamapsychiatry.2019.0943, PMID: 31141099 PMC6547104

[ref32] Hackman D. A. Gallop R. Evans G. W. Farah M. J. (2015). Socioeconomic status and executive function: developmental trajectories and mediation. Dev. Sci. 18, 686–702. doi: 10.1111/desc.12246, PMID: 25659838

[ref33] Hall P. A. Fong G. T. Epp L. J. Elias L. J. (2008). Executive function moderates the intention-behavior link for physical activity and dietary behavior. Psychol. Health 23, 309–326. doi: 10.1080/14768320701212099, PMID: 25160480

[ref34] Kail R. (1991). Development of processing speed in childhood and adolescence. Adv. Child Dev. Behav. 23, 151–185. doi: 10.1016/s0065-2407(08)60025-7 1767721

[ref35] Kail R. V. Ferrer E. (2007). Processing speed in childhood and adolescence: longitudinal models for examining developmental change. Child Dev. 78, 1760–1770. doi: 10.1111/j.1467-8624.2007.01088.x, PMID: 17988319

[ref36] Kane M. J. Brown L. H. McVay J. C. Silvia P. J. Myin-Germeys I. Kwapil T. R. (2007). For whom the mind wanders, and when: an experience-sampling study of working memory and executive control in daily life. Psychol. Sci. 18, 614–621. doi: 10.1111/j.1467-9280.2007.01948.x, PMID: 17614870

[ref37] Karr J. E. Areshenkoff C. N. Rast P. Hofer S. M. Iverson G. L. Garcia-Barrera M. A. (2018). The unity and diversity of executive functions: a systematic review and re analysis of latent variable studies. Psychol. Bull. 144, 1147–1185. doi: 10.1037/bul0000160, PMID: 30080055 PMC6197939

[ref38] Kopetz C. Woerner J. I. MacPherson L. Lejuez C. W. Nelson C. A. Zeanah C. H.. (2019). Early psychosocial deprivation and adolescent risk-taking: the role of motivation and executive control. J. Exp. Psychol. Gen. 148, 388–399. doi: 10.1037/xge0000486, PMID: 30221961 PMC7181402

[ref39] Ladouceur C. D. (2020). COVID-19 Adolescent Symptom & Psychological Experience Questionnaire. NIH - National Library of Medicine. Available at: https://www.nlm.nih.gov/dr2/CASPE_AdolSelfReport_Qualtrics.pdf (Accessed October 13, 2022)

[ref40] Liang Z. Mazzeschi C. Delvecchio E. (2021). The impact of parental stress on Italian adolescents’ internalizing symptoms during the COVID-19 pandemic: a longitudinal study. Int. J. Environ. Res. Public Health 18:8074. doi: 10.3390/ijerph18158074, PMID: 34360369 PMC8345594

[ref41] Lund J. I. Toombs E. Radford A. Boles K. Mushquash C. (2020). Adverse childhood experiences and executive function difficulties in children: a systematic review. Child Abuse Negl. 106:104485. doi: 10.1016/j.chiabu.2020.104485, PMID: 32388225

[ref42] Madhushanthi H. J. Wimalasekera S. W. Goonewardena C. S. E. Amarasekara A. A. T. D. Lenora J. (2018). Socioeconomic status is a predictor of neurocognitive performance of early female adolescents. Int. J. Adolesc. Med. Health 32. doi: 10.1515/ijamh-2018-0024 29897881

[ref43] Magson N. R. Freeman J. Y. A. Rapee R. M. Richardson C. E. Oar E. L. Fardouly J. (2020). Risk and protective factors for prospective changes in adolescent mental health during the COVID-19 pandemic. J. Youth Adolesc. 50, 44–57. doi: 10.1007/s10964-020-01332-9, PMID: 33108542 PMC7590912

[ref44] Márquez-Caraveo M. E. Rodríguez-Valentín R. Pérez-Barrón V. Vázquez-Salas R. A. Sánchez-Ferrer J. C. de Castro F.. (2021). Children and adolescents with neurodevelopmental disorders show cognitive heterogeneity and require a person-centered approach. Sci. Rep. 11:18463. doi: 10.1038/s41598-021-97551-6, PMID: 34531454 PMC8445997

[ref46] Monninger M. Pollok T. M. Aggensteiner P. M. Kaiser A. Reinhard I. Hermann A.. (2022). Coping under stress: prefrontal control predicts stress burden during the COVID-19 crisis. Eur. Neuropsychopharmacol. 56, 13–23. doi: 10.1016/j.euroneuro.2021.11.007, PMID: 34894621 PMC8606266

[ref47] Mulder H. Pitchford N. J. Marlow N. (2010). Processing speed and working memory underlie academic attainment in very preterm children. Arch. Dis. Child Fetal Neonatal Ed. 95, F267–F272. doi: 10.1136/adc.2009.167965, PMID: 20488865

[ref48] Muris P. Merckelbach H. Meesters C. van den Brand K. (2002). Cognitive development and worry in normal children. Cogn. Ther. Res. 26, 775–787. doi: 10.1023/A:1021241517274

[ref004] Natsuaki M. N. Klimes-Dougan B. Ge X. Shirtcliff E. A. Hastings P. D. Zahn-Waxler C. (2009). Early pubertal maturation and internalizing problems in adolescence: Sex differences in the role of cortisol reactivity to interpersonal stress. J. Clin Child Adolesc Psychol. 38, 513–524. doi: 10.1080/15374410902976320, PMID: 20183638 PMC3061854

[ref49] Nigg J. T. Jester J. M. Stavro G. M. Ip K. I. Puttler L. I. Zucker R. A. (2017). Specificity of executive functioning and processing speed problems in common psychopathology. Neuropsychology 31, 448–466. doi: 10.1037/neu0000343, PMID: 28094999 PMC5408314

[ref50] Nugiel T. Roe M. A. Engelhardt L. E. Mitchell M. E. Zheng A. Church J. A. (2020). Pediatric ADHD symptom burden relates to distinct neural activity across executive function domains. NeuroImage Clin 28:102394. doi: 10.1016/j.nicl.2020.102394, PMID: 32971467 PMC7511724

[ref51] Ojeda N. Sánchez P. Peña J. Elizagárate E. Yoller A. B. Gutiérrez-Fraile M.. (2012). Un modelo explicativo de la calidad de vida en la esquizofrenia: el papel de la velocidad de procesamiento y los síntomas negativos. Actas Espanolas de Psiquiatria 40, 10–18. PMID: 22344491

[ref52] Perica M. I. Ravindranath O. Calabro F. J. Foran W. Luna B. (2021). Hippocampal-prefrontal connectivity prior to the COVID-19 pandemic predicts stress reactivity. Biol Psychiatry Glob Open Sci 1, 283–290. doi: 10.1016/j.bpsgos.2021.06.010, PMID: 34849503 PMC8612769

[ref53] Porter B. M. Douglas I. J. Larguinho T. L. Aristizabal M. Mitchell M. E. Roe M. A.. (2021). Examination of pre-pandemic measures on youth well-being during early stages of the COVID-19 pandemic. Biol Psychiatry Glob Open Sci 1, 252–260. doi: 10.1016/j.bpsgos.2021.08.003, PMID: 34549203 PMC8446746

[ref54] Puig O. Penadés R. Baeza I. Sánchez-Gistau V. de la Serna E. Fonrodona L.. (2012). Processing speed and executive functions predict real-world everyday living skills in adolescents with early-onset schizophrenia. Eur. Child Adolesc. Psychiatry 21, 315–326. doi: 10.1007/s00787-012-0262-0, PMID: 22354179

[ref55] R Core Team (2021). R: A Language and Environment for Statistical Computing. R Foundation for Statistical Computing, Vienna, Austria.

[ref56] Racine N. McArthur B. A. Cooke J. E. Eirich R. Zhu J. Madigan S. (2021). Global prevalence of depressive and anxiety symptoms in children and adolescents during COVID-19. JAMA Pediatr. 175, 1142–1150. doi: 10.1001/jamapediatrics.2021.2482, PMID: 34369987 PMC8353576

[ref57] Robson D. A. Allen M. S. Howard S. J. (2020). Self-regulation in childhood as a predictor of future outcomes: a meta-analytic review. Psychol. Bull. 146, 324–354. doi: 10.1037/bul0000227, PMID: 31904248

[ref003] Roebers C. M. (2017). Executive function and metacognition: Towards a unifying framework of cognitive self-regulation. Developmental Rev. 45, 31–51. doi: 10.1016/j.dr.2017.04.001

[ref59] Salthouse T. A. (1996). The processing-speed theory of adult age differences in cognition. Psychol. Rev. 103, 403–428. doi: 10.1037/0033-295x.103.3.403 8759042

[ref60] Snyder H. R. Miyake A. Hankin B. L. (2015). Advancing understanding of executive function impairments and psychopathology: bridging the gap between clinical and cognitive approaches. Front. Psychol. 6:328. doi: 10.3389/fpsyg.2015.00328, PMID: 25859234 PMC4374537

[ref61] Songco A. Hudson J. L. Fox E. (2020). A cognitive model of pathological worry in children and adolescents: a systematic review. Clin. Child. Fam. Psychol. Rev. 23, 229–249. doi: 10.1007/s10567-020-00311-7, PMID: 31989444 PMC7192867

[ref62] Sung Y. Choi E. (2021). The reciprocal longitudinal relationship between executive dysfunction and happiness in Korean children. Int. J. Environ. Res. Public Health 18:7764. doi: 10.3390/ijerph18157764, PMID: 34360057 PMC8345533

[ref63] Tang S. Xiang M. Cheung T. Xiang Y.-T. (2021). Mental health and its correlates among children and adolescents during COVID-19 school closure: the importance of parent child discussion. J. Affect. Disord. 279, 353–360. doi: 10.1016/j.jad.2020.10.016, PMID: 33099049 PMC7550131

[ref64] Thornton C. P. Ruble K. Jacobson L. A. (2021). Beyond risk-based stratification: impacts of processing speed and executive function on adaptive skills in adolescent and young adult cancer survivors. J. Adolesc. Young Adult Oncol. 10, 288–295. doi: 10.1089/jayao.2020.0059, PMID: 32668177

[ref65] Toplak M. E. West R. F. Stanovich K. E. (2012). Practitioner review: do performance-based measures and ratings of executive function assess the same construct? J. Child Psychol. Psychiatry 54, 131–143. doi: 10.1111/jcpp.12001, PMID: 23057693

[ref66] Trossman R. Spence S.-L. Mielke J. G. McAuley T. (2021). How do adverse childhood experiences impact health? Exploring the mediating role of executive functions. Psychol. Trauma Theory Res. Pract. Policy 13, 206–213. doi: 10.1037/tra0000965, PMID: 32940523

[ref67] Tsai N. Jaeggi S. M. Eccles J. S. Atherton O. E. Robins R. W. (2020). Predicting late adolescent anxiety from early adolescent environmental stress exposure: cognitive control as mediator. Front. Psychol. 11:1838. doi: 10.3389/fpsyg.2020.01838, PMID: 32849080 PMC7432129

[ref68] Unsworth N. Robison M. K. (2016). The influence of lapses of attention on working memory capacity. Mem. Cogn. 44, 188–196. doi: 10.3758/s13421-015-0560-0, PMID: 26450588

[ref69] Wagner C. A. Alloy L. B. Abramson L. Y. (2014). Trait rumination, depression, and executive functions in early adolescence. J. Youth Adolesc. 44, 18–36. doi: 10.1007/s10964-014-0133-8, PMID: 24839132 PMC4236277

[ref72] Weissman D. G. Rodman A. M. Rosen M. L. Kasparek S. Mayes M. Sheridan M. A.. (2021). Contributions of emotion regulation and brain structure and function to adolescent internalizing problems and stress vulnerability during the COVID-19 pandemic: a longitudinal study. Biol Psychiatry Glob Open Sci 1, 272–282. doi: 10.1016/j.bpsgos.2021.06.001, PMID: 34901918 PMC8643098

[ref73] Zetsche U. Joormann J. (2011). Components of interference control predict depressive symptoms and rumination cross-sectionally and at six months follow-up. J. Behav. Ther. Exp. Psychiatry 42, 65–73. doi: 10.1016/j.jbtep.2010.06.001, PMID: 20650447

[ref74] Zhang S. Y. Qiu S. W. Pan M. R. Zhao M. J. Zhao R. J. Liu L.. (2021). Adult ADHD, executive function, depressive/anxiety symptoms, and quality of life: a serial two-mediator model. J. Affect. Disord. 293, 97–108. doi: 10.1016/j.jad.2021.06.020, PMID: 34175595

[ref75] Zhang C. Ye M. Fu Y. Yang M. Luo F. Yuan J.. (2020). The psychological impact of the COVID-19 pandemic on teenagers in China. J. Adolesc. Health 67, 747–755. doi: 10.1016/j.jadohealth.2020.08.026, PMID: 33041204 PMC7543885

[ref76] Zhang Y. Zhang X. Zhang L. Guo C. (2019). Executive function and resilience as mediators of adolescents’ perceived stressful life events and school adjustment. Front. Psychol. 10:446. doi: 10.3389/fpsyg.2019.00446, PMID: 30873099 PMC6403185

[ref77] Zhou S.-J. Zhang L.-G. Wang L.-L. Guo Z.-C. Wang J.-Q. Chen J.-C.. (2020). Prevalence and socio-demographic correlates of psychological health problems in Chinese adolescents during the outbreak of COVID-19. Eur. Child Adolesc. Psychiatry 29, 749–758. doi: 10.1007/s00787-020-01541-4, PMID: 32363492 PMC7196181

