# Supplementary material for: Youth pre-pandemic executive function relates to year one COVID-19 difficulties
Source: Front Psychol. 2023 Apr 20;14:1033282. doi: 10.3389/fpsyg.2023.1033282 (PMC10156991; doi:10.3389/fpsyg.2023.1033282)
Supplement: Supplementary file 1 [file Data_Sheet_1.pdf]

Supplement

Table 1

Demographic Information

	Early Pandemic (N = 135)	Mid-Pandemic (N = 107)
**Age**		
Mean (SD)	15.2 (3.03)	15.7 (3.02)
Median [Min, Max]	15.0 [9.45, 22.1]	15.7 [10.1, 21.7]
**Gender**		
Female	61 (45%)	52 (49%)
Male	70 (52%)	51 (48%)
Non-binary	3 (2%)	2 (2%)
Prefer not to say	1 (1%)	2 (2%)
**Race/Ethnicity**		
American Indian or Alaskan Native	3 (2%)	3 (3%)
Asian	1 (1%)	0 (0%)
Black	4 (3%)	4 (4%)
Hispanic/Latinx (Multiracial)	18 (13%)	17 (16%)
Hispanic/Latinx (not Multiracial)	14 (10%)	7 (7%)
Multiracial	6 (4%)	6 (6%)
White	88 (65%)	70 (65%)
Other	1 (1%)	0 (0%)
**Diagnosis**		
ADHD only	39 (29%)	32 (30%)
ADHD (with comorbidity)	17 (13%)	11 (10%)
Anxiety	12 (9%)	7 (7%)
ASD	2 (1%)	2 (2%)
Depression	6 (4%)	2 (2%)
Dyslexia	6 (4%)	4 (4%)
OCD (with comorbidity)	6 (4%)	4 (4%)
TS	2 (1%)	2 (2%)
No Diagnosis	63 (47%)	52 (49%)
Multiple diagnoses (2 or more)	23 (17%)	15 (14%)
**Medication**		
Diagnosed, medicated	25 (19%)	21 (20%)
Diagnosed, not medicated	45 (33%)	34 (32%)

Not diagnosed, not medicated	62 (46%)	52 (49%)
**Income**	(n=128)	(n=102)
Mean (SD)	150000 (124000)	140000 (115000)
Median [Min, Max]	120000 [0, 1000000]	120000 [0, 1000000]

Table 1 Note. Demographic information of participants broken down by COVID-19 timepoint. The age of the participants refers to their age when completing the respective COVID-19 survey. Comorbidity with ADHD and OCD included anxiety, ASD, depression, ODD, dyslexia, and/or TS. Medication status was missing from 2 participants included in COVID-19 survey timepoint 1. Analyses including the socio-economic composite measure had reduced early- (n = 129) and mid-pandemic (n = 103) sample size due to missing data.

Table 2

Pre-Pandemic Cognitive Tasks

**Domain**<br>**Task**	**Description**	**Variable**
**Updating**		
N-Back	Indicating whether the presented shape within a series matched one (1-back) or two (2-back) shaped before	Number of correct hits summed with the number of correct missed hits
Symmetry Span	Switching between two distinct segments: determining symmetry and memorizing position/sequent of cues	Mean accurate recalled squares
WISC/WAIS Digit Span	Hearing increasingly long series of number and repeating them verbally back forwards and backwards (both WISC and WAIS), and ascending order (WAIS only)	Mean accuracy in forward and backward, and (if applicable) sequencing conditions
**Switching**		
Cognitive Flexibility	Alternating between matching an image with two additional images based on shape or color.	Mean all-trials accuracy
Local-Global	Switch between two rules of verbally naming letters	Mean accuracy among incongruent trials
Connections	Alternate between connecting numbers in ascending orders and letters in alphabetical order	Mean accuracy in incongruent trials
**Inhibition**		
Animal Stroop	Correctly naming animals despite being presented with competing information	Mean accuracy within incongruent trials

Stop Signal	Avoid indicating arrow direction when cue was present	Reverse coded stop signal reaction time (SSRT)
**Processing Speed**		
Pattern and Letter Comparisons	Determine whether two patterns or letter sequences were distinct or the same	Mean total seconds spent per trial divided by the mean of correct response across both trials
WISC-IV/WAIS-II Symbol Search	Indicate whether there were matching geometric figures within two series distinct (WISC-IV and WAIS-II) and signal repeated figure (WAIS-II only)	Total number of correct answers divided from the total task time

Note. N-Back, Symmetry Span, WISC/WAIS Digit Span, Cognitive Flexibility, Local-Global, Connections, Animal Stroop, Stop Signal, Pattern and Letter Comparisons, and WISC-IV/ WAIS-II Symbol Search task descriptions and scoring.

Table 3

Cronbach's Alpha of Mid-pandemic Experience Sub-scales

	Emotional Difficulties	Cognitive Difficulties	Social Interactions
Cronbach's alpha	0.85	0.54	0.66

Note. Cronbach's alpha for the mid-pandemic COVID-19 functioning scales.

Table 4

EF and Processing Speed as Predictors of Early-pandemic Experience

	Emotional Difficulties				Cognitive Difficulties				Social Interactions			
	β	*p*	R^2	Adjusted R^2	*β*	*p*	R^2	Adjusted R^2	*β*	*p*	R^2	Adjusted R^2
Common EF	-0.0003	1.00	0.042	0.028	-0.049	1.00	0.023	0.0079	0.11	1.00	0.056	0.041
Inhibition	-0.069	0.63	0.044	0.029	0.11	0.63	0.026	0.011	0.095	0.63	0.056	0.042
Updating	0.047	0.99	0.043	0.029	-0.15	0.88	0.030	0.016	-0.0025	0.99	0.053	0.039
Switching	0.015	0.91	0.042	0.028	-0.039	0.91	0.023	0.0079	0.10	0.91	0.058	0.043
PS	0.086	0.51	0.042	0.027	0.10	0.51	0.023	0.0082	-0.27	0.11	0.077	0.063

EF and Processing Speed as Predictors of Mid-pandemic Experience

	Emotional Difficulties				Cognitive Difficulties				Social Interactions			
	β	*p*	R^2	Adjusted R^2	*β*	*p*	R^2	Adjusted R^2	*β*	*p*	R^2	Adjusted R^2
Common EF		—				—			0.34	0.074	0.046	0.028
Inhibition	0.20	0.58	0.11	0.097	0.042	0.79	0.39	0.021	0.13	0.60	0.023	0.0044
Updating		—				—			0.18	0.20	0.032	0.013

Note. These analyses included age as a covariate and p-values were FDR-corrected. EF = executive function; PS = processing speed.

Table 5

Family Bootstrapped Analysis of Mid-Pandemic Timepoint (n=68)

	Emotional Difficulties	Cognitive Difficulties	Social Interactions
	Mp	*Mp*	*Mp*
Common EF	0.05	0.16	—
Updating	0.08	0.09	—
Switching	0.11	0.16	0.15
PS	0.13	0.09	0.24

Note. Mean p-values (Mp) of bootstrapping by 1000 times selecting 1 random child per family and running the same regressions as with the whole sample (n=107). All regressions controlled for age. PS = processing speed.

Table 6

Exploratory Variables and COVID-Era Functioning

	Emotional Difficulties				Cognitive Difficulties				Social Interactions			
**Gender**												
					t	*p*	*df*		*t*	*p*	*df*	
Early Pandemic	—				1.90	0.089	129		0.21	0.83	129	
Mid-Pandemic	—				0.19	0.85	101		1.30	0.29	101	
**SES**												
	β	*p*	R^2	*Adjusted* R^2	*β*	*p*	R^2	*Adjusted* R^2	*β*	*p*	R^2	*Adjusted* R^2
Early Pandemic	-0.046	0.66	0.046	0.030	-0.19	0.36	0.050	0.034	0.045	0.66	0.057	0.041
Mid- Pandemic	0.048	0.74	0.11	0.086	0.11	0.74	0.071	0.050	0.10	0.74	0.021	-0.0011
**MHB**												
	β	*p*	R^2	*Adjusted* R^2	*β*	*p*	R^2	*Adjusted* R^2	*β*	*p*	R^2	*Adjusted* R^2
Mid-Pandemic	-0.47	0.59	0.10	0.084	-1.21	0.5	0.056	0.038	-0.60	0.59	0.021	0.0019
**Time interval**												
	β	*p*	R^2	*Adjusted* R^2	*β*	*p*	*R*	R^2	*β*	*p*	*R*	R^2
Early Pandemic	-0.046	0.69	0.043	0.029	0.11	0.52	0.029	0.014	0.19	0.30	0.072	0.058
Mid- Pandemic	-0.036	0.97	0.10	0.083	0.0054	0.97	0.039	0.020	0.088	0.97	0.021	0.0024

Note. These analyses were FDR-corrected. "Time interval" refer to the time gap between pre-pandemic and the respective COVID-era data. MHB = mental health burden. SES = socioeconomic composite score.

Table 7

EF and Processing Speed as Predictors of Mid-pandemic Experience, Controlling for Exploratory Covariates

	Emotional Difficulties				Cognitive Difficulties				Social Interactions			
**Gender**												
	β	*p*	R^2	Adjusted R^2	*β*	*p*	R^2	Adjusted R^2	*β*	*p*	R^2	Adjusted R^2
Common EF	0.48	0.014*	0.24	0.21	0.49	0.014*	0.10	0.076	—			
Updating	0.32	0.018*	0.23	0.20	0.36	0.018*	0.10	0.077	—			
Switching	0.31	0.031*	0.22	0.19	0.41	0.011*	0.12	0.091	—			
**SES**												
	β	*p*	R^2	Adjusted R^2	*β*	*p*	R^2	Adjusted R^2	*β*	*p*	R^2	Adjusted R^2
Common EF	0.59	0.0058*	0.17	0.14	0.53	0.011*	0.13	0.096	—			
Updating	0.37	0.011*	0.16	0.13	0.38	0.011*	0.13	0.10	—			
Switching	0.41	0.0059*	0.17	0.15	0.45	0.0059**	0.17	0.14	0.30	0.046*	0.059	0.028
PS	-0.30	0.034*	0.15	0.12	-0.37	0.034*	0.15	0.12	0.31	0.034*	0.073	0.042
**MHB**												
	β	*p*	R^2	Adjusted R^2	*β*	*p*	R^2	Adjusted R^2	*β*	*p*	R^2	Adjusted R^2
Common EF	0.53	0.012*	0.17	0.15	0.49	0.015*	0.11	0.089	—			
Updating	0.34	0.018*	0.13	0.10	0.36	0.018*	0.11	0.083	—			
Switching	0.37	0.012*	0.16	0.14	0.42	0.01*	0.13	0.11	0.27	0.062	0.053	0.026
PS	-0.28	0.045*	0.13	0.10	0.34	0.045*	0.11	0.083	0.31	0.045*	0.059	0.031
**Time Interval**												
	β	*p*	R^2	Adjusted R^2	*β*	*p*	R^2	Adjusted R^2	*β*	*p*	R^2	Adjusted R^2
Common EF	0.55	0.0066**	0.17	0.15	0.56	0.0066**	0.11	0.086	—			
Updating	0.36	0.014*	0.16	0.13	0.41	0.011*	0.11	0.089	—			
Switching	0.38	0.010*	0.16	0.14	0.46	0.0048**	0.13	0.10	0.31	0.033*	0.064	0.036
PS	-0.28	0.043*	0.13	0.10	-0.37	0.031*	0.99	0.073	-0.33	0.031*	0.066	0.038

Note. These analyses also included age as a covariate. Only originally significant relations were tested for robustness to these covariates. "Time interval" refer to the gap time between the acquisition of pre-pandemic and mid-pandemic data. EF = executive function; PS = processing speed; SES = socioeconomic composite score; MHB = mental health burden. ***$p < 0.01$, *$p < 0.05$, FDR-corrected*
